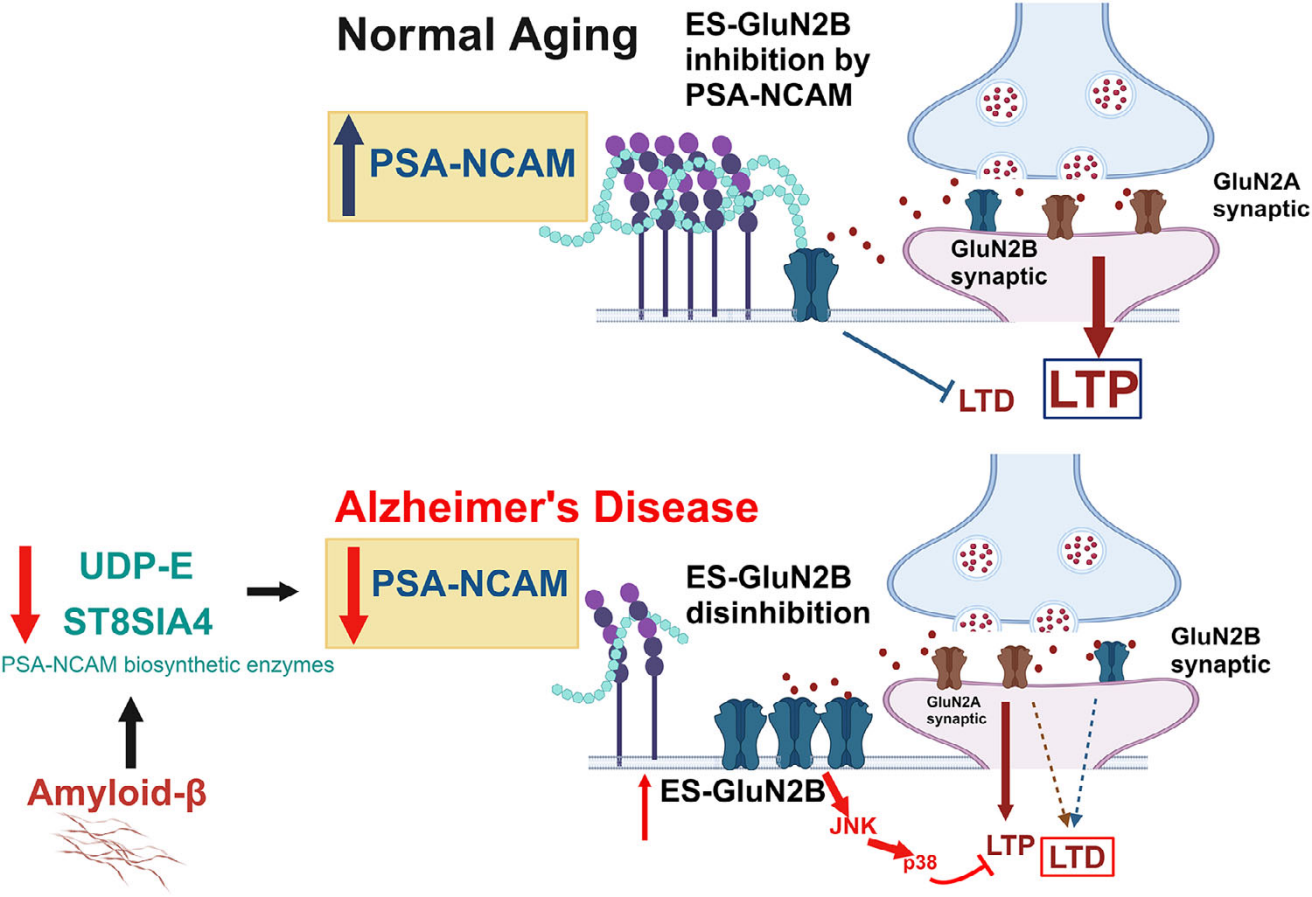# Spatiotemporal differential regulation of extrasynaptic GluN2B receptor subunits and PSA‐NCAM in brain aging and Alzheimer's disease

**DOI:** 10.1002/alz70855_102267

**Published:** 2025-12-23

**Authors:** Oghenetega E Imiruaye, Isis Perez, Brian Carson, Christian Crouzet, Jerome Garcia, Derick Han, Subhrajit Bhattacharya

**Affiliations:** ^1^ Keck Graduate Institute, Claremont, CA, USA; ^2^ University of California, Irvine, Irvine, CA, USA; ^3^ University of La Verne, La Verne, CA, USA

## Abstract

**Background:**

N‐methyl‐D‐aspartate receptors (NMDARs) are critical for synaptic transmission, with GluN2B subunits playing key roles in synaptic plasticity. Extrasynaptic GluN2Bs (ES‐GluN2Bs) activate long‐term depression (LTD) pathways, potentially promoting dementia in Alzheimer's disease (AD). Polysialylation of neural cell adhesion molecule (NCAM) to PSA‐NCAM by ST8‐α‐N‐acetyl‐neuraminide‐α‐2,8‐sialyltransferase‐4 (ST8Sia4) and UDP‐N‐acetylglucosamine‐2‐epimerase (UDP‐E) regulates synaptic remodeling and inhibits ES‐GluN2B activity physiologically. However, the spatiotemporal dynamics of ES‐GluN2Bs and PSA‐NCAM in brain aging versus AD and how Aβ, a pathological hallmark of AD, affects these proteins remain unclear.

**Method:**

We examined expression levels of NMDAR subunits (GluN2A, GluN2B), ES‐GluN2Bs, NCAM, and PSA‐NCAM in young and old Tg2576 AD mice and wild‐type (WT) controls across the cortex, prefrontal cortex, hippocampus, and midbrain using immunoblotting and pull‐down assays. After this, we investigated the neurochemical effects of varying concentrations of Aβ treatment on ST8Sia4, PSA‐NCAM, and UDP‐E expression via protein and mRNA quantification in IMR‐32 neuroblastoma cells.

**Result:**

Aging reduced overall GluN2B expression in both WT and AD mice (47–51%, n≥4) while increasing GluN2A expression (up to 85%, n≥4). ES‐GluN2B levels were significantly elevated in AD mice (2–3‐fold, n≥4), but unchanged in WT mice. PSA‐NCAM expression was downregulated in AD mice (by 43–58%, n≥4), particularly in the hippocampus and prefrontal cortex, while increasing with normal aging (up to 2‐fold, n≥4). Analysis of protein and mRNA expression levels following Aβ treatment in IMR‐32 cells revealed significant downregulation (up to 60%) in ST8Sia4, PSA‐NCAM, and UDP‐E across all concentrations.

**Conclusion:**

Our findings demonstrate AD‐specific increases in ES‐GluN2B expression and a significant downregulation in PSA‐NCAM levels, distinguishing AD from normal aging, potentially driven by Aβ‐induced downregulation of biosynthetic enzymes ST8Sia4 and UDP‐E. This underscores a potential link between PSA‐NCAM expression and Aβ activity in AD, as well as possible therapeutic targets for AD intervention.